# Chronic Osteomyelitis of the Jaw: Pivotal Role of Microbiological Investigation and Multidisciplinary Management—A Case Report

**DOI:** 10.3390/antibiotics11050568

**Published:** 2022-04-24

**Authors:** Quentin Lucidarme, Delphine Lebrun, Véronique Vernet-Garnier, Joey Le Gall, Saïdou Diallo, Cédric Mauprivez, Stéphane Derruau

**Affiliations:** 1 Pôle de Médecine Bucco-Dentaire, Service de Chirurgie Orale, Centre Hospitalier Universitaire de Reims, 51092 Reims, France; quentin.lucidarme@gmail.com (Q.L.); legalljoey@gmail.com (J.L.G.); cmauprivez@chu-reims.fr (C.M.); 2 Service de Médecine Interne et Maladies Infectieuses, Centre Hospitalier Intercommunal Nord Ardennes, 08000 Charleville-Mézières, France; dlebrun@chu-reims.fr; 3 Centre de Référence des Infections Ostéo-Articulaires Complexes (CRIOAC Inter-Région Nord-Est), Centre Hospitalier Universitaire de Reims, 51092 Reims, France; vvernetgarnier@chu-reims.fr (V.V.-G.); sdiallo@chu-reims.fr (S.D.); 4 Service de Bactériologie, Centre Hospitalier Universitaire de Reims, 51092 Reims, France; 5 Service d’Orthopédie-Traumatologie, Centre Hospitalier Universitaire de Reims, 51092 Reims, France; 6 Laboratoire EA-4691 Biomatériaux et Inflammation en Site Osseux, UFR de Pharmacie, Université de Reims Champagne-Ardenne, 51096 Reims, France; 7 Laboratoire BioSpecT EA-7506, UFR de Pharmacie, Université de Reims Champagne-Ardenne, 51096 Reims, France

**Keywords:** osteomyelitis, jaw, child, microbiology, MALDI-TOF analysis, case report

## Abstract

A 15-year-old girl with a history of recurrent painful orofacial swelling was diagnosed on the basis of clinical findings, histopathological examination and imaging modalities as having primary chronic osteomyelitis of the jaw. Initial microbiological samples were performed but were inconclusive. She received multiple empirical antibiotic therapies and NSAIDs for 3 years without complete remission. Only MALDI-TOF (Matrix-Assisted Laser Desorption/Ionization–Time Of Flight) analysis after additional multiple microbiological bone samples with adequate techniques yielded the final diagnosis of bacterial chronic osteomyelitis of the jaw. Its management requires a multidisciplinary approach, involving oral and maxillofacial surgeons, infectiologists and microbiologists, to limit treatment failure. Antibiotic therapy without surgery for 6 months achieved the complete radiographic resolution of the CBCT (Cone Beam Computed Tomography) and the normalization of laboratory tests. After 2 years of follow-up, no relapse had been reported. Modern microbiological investigation and sampling techniques are critical for the accurate diagnosis and management of osteomyelitis of the jaw, especially in unusual and clinically misleading forms of this infection.

## 1. Introduction

Jawbone infections present several challenges to dentists and oro-maxillofacial surgeons despite recent advances in diagnosis, surgical management and antimicrobial therapy [1].

Osteomyelitis is defined as an inflammation of cortical and cancellous bone, which is mainly caused by bacteria or fungi. Jawbone infections usually progress from an uncontrolled dental problem by simple contiguous spread (e.g., pulpal and periodontal infections) or by direct inoculation from trauma or surgery (e.g., tooth extractions, oral mucosal wounds and maxillofacial fractures). Less frequently, hematogenous spread from bacteremia or a remote infectious site may be considered causal, especially in acute osteomyelitis in infants and children [2].

Although several classifications of osteomyelitis of the jaw (OJ) have been proposed, the Zurich classification system is currently the most widely used [2]. OJ is first described according to duration, either acute or chronic, clinical features and imaging. Subclassification is achieved through the histopathology, presumed etiology and pathogenesis of the disease.

Acute and secondary chronic OJ are true infections at different stages of the same disease. There are several underlying bone pathologies and physiological conditions that affect bone homeostasis and local vascularity, facilitating bone infections in the jaw. Diabetes mellitus, irradiation of the orofacial sphere and bisphosphonate therapy are known to be risk factors [2]. In cases where necrotic bone is exposed to the oral cavity, the microorganisms found are considered as contaminants coming from oral microbiota [3].

In the absence of pus, fistula or sequester formation, the diagnosis of bone infection may be difficult. Primary chronic osteomyelitis (PCO) is suspected when no obvious association with bone infection is shown, and often based on the exclusion of other bone diseases [1,2,4]. Chronic non-bacterial osteomyelitis is an uncommon bone disease and is rarely confined to the jaw [4]. PCO mainly includes SAPHO syndrome (synovitis, acne, pustulosis, hyperostosis and osteitis), chronic recurrent multifocal osteomyelitis (CMRO) and juvenile mandibular chronic osteomyelitis (JMCO) [1,2,4]. The pathogenesis of these pathological entities (SAPHO, CMRO and JMCO) remains largely unknown, despite the development of some hypotheses [4]. PCO may be due to an altered immune response to unidentified infectious sources with a low virulence.

In this clinical case, we report the management of a case of osteomyelitis affecting the mandible in a child with an incorrect initial diagnosis related to a lack of adequate microbiological investigation.

## 2. Case Report

A 15-year-old girl from Kosovo was referred by her physician to the Oral Surgery Department of Reims University Hospital for recurrent painful orofacial swelling in June 2019.

The young patient had a 3-year history of chronic osteomyelitis of the jaw. Imaging examination, including a CT scan, and bone biopsy had been performed to support the diagnosis of a primary chronic osteomyelitis. Indeed, histopathological examination, excluding malignant pathology, had shown non-specific signs of chronic bone inflammation, and microbiological analysis had failed to detect the growth of specific bacteria. Between July 2016 and June 2019, recurring inflammatory episodes had been treated with short-term antibiotics (amoxicillin and co-amoxiclav) and non-steroidal anti-inflammatory drugs (NSAIDs; ibuprofen^®^) with a partial remission of symptoms. The rest of medical history was unremarkable.

**Figure 1:**
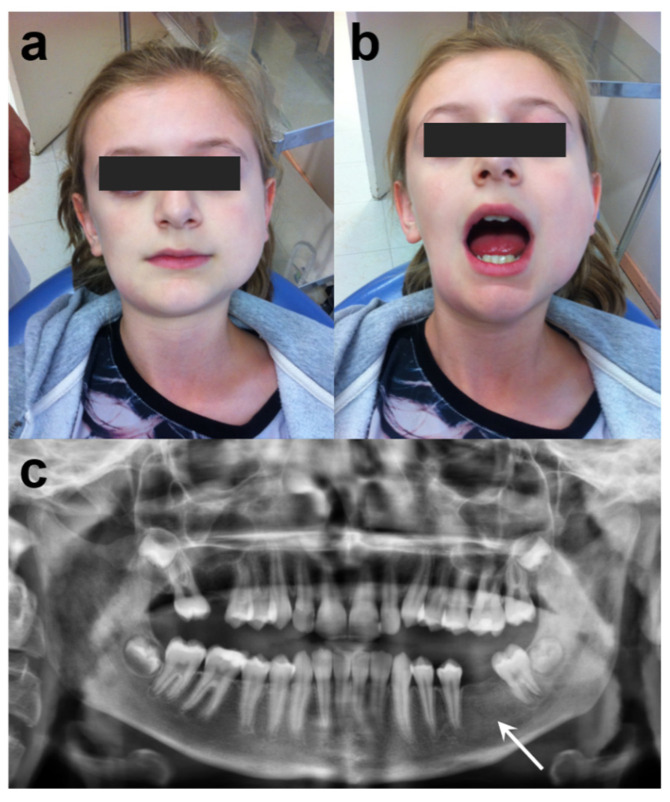
Initial presentation of patient: (**a**) frontal view of the patient, (**b**) limited mouth opening of 25 mm inter-incisor distance, (**c**) panoramic radiograph showing diffuse radiopacity in the left mandibular bone (white arrow).

Clinical examination revealed facial asymmetry with the enlargement of the left mandible, and trismus (Figure 1). A firm and painful swelling in the right forehead and lower mandibular angle was detected by extraoral palpation. No fever, regional lymph nodes, arthritis or skin lesions had been reported. Intraoral examination revealed the loss of teeth #16 and #36. No carious lesions of the dentition were observed. There were no periodontitis or signs of infections in the mouth. Initial panoramic radiograph demonstrated diffuse radiopacities in the left mandibular ramus (Figure 1).

A slight increase in white blood cells (WBCs) (11,500/µL [range, 3900–9800/µL) and C-reactive protein (CRP) (11.6 mg/dL [range, 0.00–0.50 mg/dL]) were noted.

**Figure 2:**
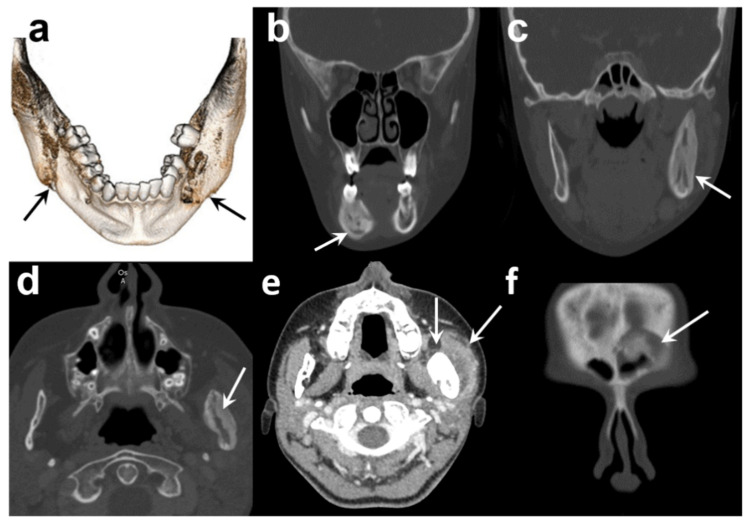
Diagnostic CT scan of the patient: (**a**) 3D reconstruction of mandible, (**b**) coronal bone-window section of sclerosis and osteolytic in right corpus of the mandible, (**c**,**d**) coronal and axial bone window showing an enlargement of left ascending ramus with osteosclerosis with few areas of osteolysis, (**e**) axial section in soft tissue window revealing a swelling of the left masseter muscle, (**f**) coronal bone-window section showing an irregular area of sclerosis involving the edge of the left frontal sinus.

**Figure 3:**
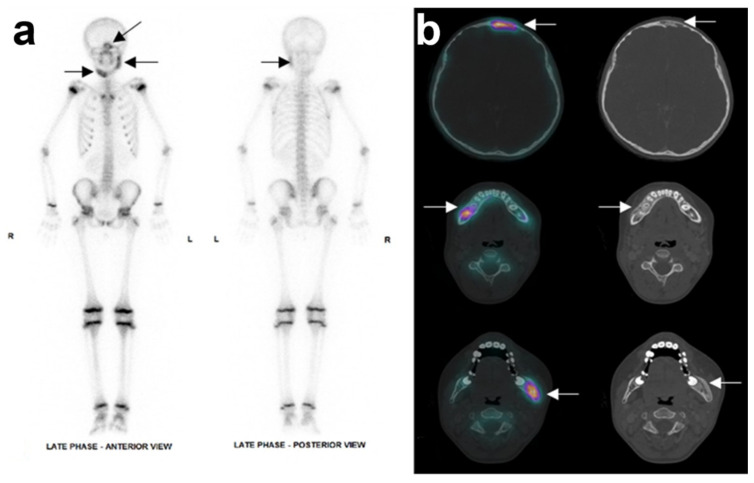
Nuclear X-ray of the patient’s initial state: (**a**) Whole-body bone scintigraphy showed three areas of increased uptake (hot spot) in the facial skeleton: in the body of the right mandible, in the angle and ascending ramus of the left mandible, and in right frontal sinus. (**b**) SPECT registered with CT scan displaying, from top to bottom line, the increased uptake in frontal sinus, right and left mandible, respectively. SPECT: single photon emission computed tomography.

A new CT scan showed that both sides of the mandible were affected, including the right corpus and left ascending ramus, leading to an expansive deformity of the bone. No sequestration was found. Another affected area was detected in the right frontal bone (Figure 2). A bone SPECT (single photon emission computed tomography) registered with this CT scan confirmed the location and areas of metabolic activity corresponding to areas of mandibular and frontal osteosclerosis and osteolysis. No other bone lesions were identified (Figure 3).

Histological biopsies were again performed under local anesthesia in the affected mandibular areas, particularly, on the right mandibular angle, corresponding clinically to the most recurrently inflammatory region and radiologically to the most representative medullary area defined in the preoperative cone beam computed tomography (CBCT). These biopsies were conducted one after another, each time using a clean and sterile kit of instruments (using bone trephine and clamps) before being stored in sterile dry containers. Immediately after harvesting all the samples, the site was largely irrigated with sterile NaCl solution before closing the vestibular mucoperiosteal flap. Histopathology ruled out childhood malignant tumors (osteosarcoma), bone diseases (e.g., fibrous dysplasia, ossifying fibroma, Paget disease and histiocytosis), and showed non-specific bone inflammation. Microbiological bone samples (*n* = 6), in fresh state, were collected via an intraoral approach with a strict protocol to avoid oral contamination. Biopsy samples were analyzed for aerobic and anaerobic bacterial cultures, and fungal and mycobacterial cultures. *Actinomyces oris*, *Streptococcus gordonii* and *Streptococcus vestibularis* were isolated from 4 of the 6 samples. Medical data referred to Champagne-Ardenne CRIOAC (Centre de Référence des Infections Ostéoarticulaires complexes) to improve bacteriological analysis/diagnosis and therapeutic decision making.

**Figure 4:**
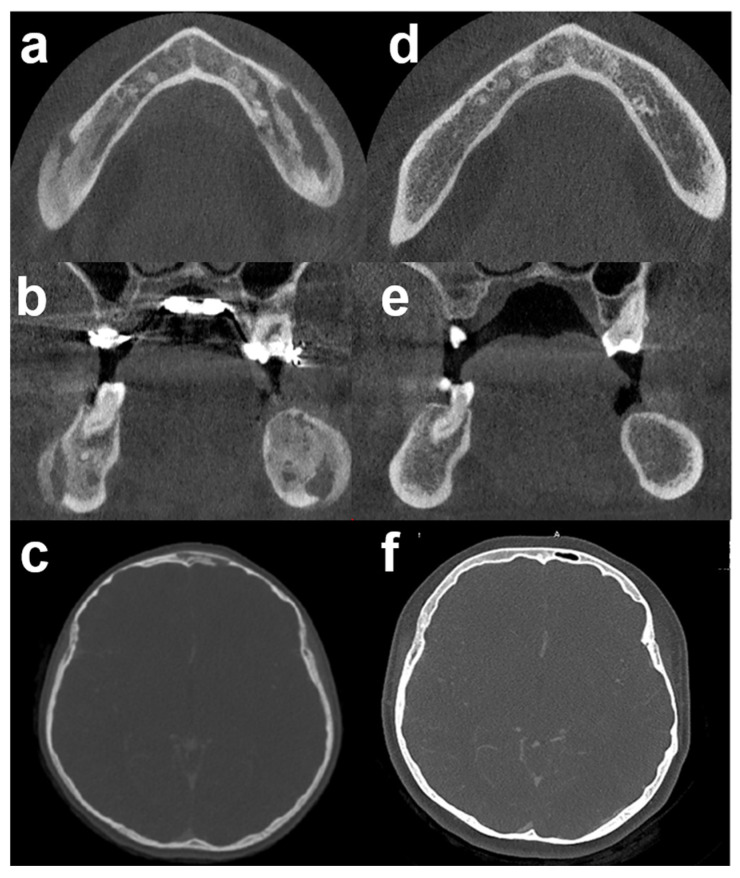
3D computed tomography before and after antibiotic therapy: (**a**–**c**) CBCT/CT scan before antibiotic therapy. (**d**–**f**) The corresponding CBCT/CT scan, 6 months after antibiotic therapy demonstrated complete bone healing. Bone sclerosis and osteolysis (mixt pattern) disappeared in mandible (**d**,**e**) and frontal bone (**f**). CBCT: Cone Beam Computed Tomography.

Based on these findings, a final diagnosis of chronic bacterial osteomyelitis was established. We initiated a 6-month course of oral antibiotic treatment with levofloxacin (500 mg, once a day) and cotrimoxazole (= trimethoprim/sulfamethoxazole, TMP/SMX) (400 mg/80 mg, two times a day). Periodic blood tests and renal function monitoring were performed with normal results. At 3 months, the patient was symptom-free. At 6 months, CBCT/CT scans showed a normal bone structure (Figure 4). At 2 years post-antibiotic treatment, no clinical or radiological relapse had been noted.

## 3. Discussion

The clinical case of osteomyelitis of the jaw (OJ) reported in this paper perfectly illustrates that an accurate diagnosis is essential for adequate and effective management. In the absence of suggestive signs of infection, the exact etiology of OJ is challenging and appropriate treatment is often delayed [1,2].

According to the Zurich classification, primary chronic osteomyelitis (PCO) describes a heterogenous group of rare chronic non-bacterial osteomyelitis for which etiologies remain unknown [2]. However, few authors suggest an infectious etiology even if no evidence of a causal relationship between the presence of bacteria and the disease are found [3,5]. Microbial cultures fail to detect specific pathogen bacteria, and samples are often contaminated by saliva or commensal bacteria of the mouth. Predominant isolated bacteria belong to the genera *Streptococcus*, *Peptostreptococcus*, *Actinomyces* and *Cutibacterium* (formerly *Propionibacterium*) [2]. Although these findings support a non-infectious disease, histopathological features with the presence of micro-abscesses in most cases suggest an infectious etiology for PCO.

In the last decade, some studies have hypothesized that PCO is a polymicrobial low-grade infection caused by slow-growing bacteria that are difficult to isolate on standard culture, thereby explaining the failure of conventional methods in microbial identification [6,7]. Some of these opportunistic pathogens are barely cultivable in standard microbiological growth media. Currently, a wide range of molecular biology techniques are used to improve detection and identification, especially uncultivable and slow-growing bacteria [8]. In addition to classical biochemical tests and genomic approaches (e.g., polymerase chain reaction and 16S rRNA gene sequencing), mass spectrometry-based proteomics has become a method of choice for studying pathogenic bacteria. Matrix-Assisted Laser Desorption/Ionization–Time Of Flight (MALDI-TOF) is a type of a mass spectrometry used in many microbiological laboratories, allowing the rapid identification of bacterial pathogens and the detection of antibacterial resistance [9].

In this case, the initial diagnosis was incorrect because no dental focus had been identifiable, and the first microbiological outcomes were non-conclusive. A more detailed history revealed that dental decays had caused the loss of teeth #16 and #36. The absence of clinical response to treatment with NSAIDs and short-term probabilistic antibiotic therapy led us to reconsider the initial diagnosis. PCO (non-bacterial OJ) may be confused with non-suppurative secondary chronic osteomyelitis (bacterial OJ) [2]. Microbiological investigation is essential to avoid a misclassification of osteomyelitis and to establish differential and definite diagnosis.

Multiple bone biopsies were taken again from the medullary spaces. *Actinomyces oris*, *Streptococcus gordonii* and *Streptococcus vestibularis* were identified using MALDI Brüker’s Biotyper-specific database. Actinomyces and Streptococcus species are Gram-positive, anaerobic and facultative anaerobic bacilli and cocci, respectively. These opportunistic oral pathogens have previously been associated with various severe infections, such as osteomyelitis, bacteremia, meningitis and infective endocarditis [10].

Chronic OJ is mostly a low-grade polymicrobial infection of dental origin. The prime opportunistic pathogenic species are Streptococci and anaerobic bacteria [11]. Less commonly, bone infections can be caused by a single pathogen. In cases of hematogenous osteomyelitis, staphylococci and enteric rods are the most common specific bacteria isolated from the blood or bone [2,11].

The sampling technique is a critical factor in performing accurate microbiological analysis. Despite its crucial role, 28% to 50% of all microbial samples can often remain negative according to the current orthopedic literature [12]. To increase the sensitivity of bone cultures, guidelines recommend a 14-day antibiotic-free period prior to bone sampling and prolonged culture (up to 12 days) to improve the isolation of low-growing or dormant organisms (e.g., small colony variants emerging from biofilm) [12]. Harvesting bone from the jaw via an intraoral approach is acceptable, provided that the protocol guarantees strict asepsis and a rigorous sampling technique [9]. A skin and oral mucosa disinfection prior to the sample and depth bone biopsies with no contact with saliva is necessary [9,13]. Bone samples, in fresh state, must be quickly transported to a microbiological laboratory (acceptable time delay less than 2 h) in a sterile tube [3]. Microbiologically, a multitude of bone samples are required to identify OJ causal bacteria from contaminant bacteria [13]. At least five bone samples are required, and each sample must be harvested with unique sterile instruments. The infection is considered true when (i) at least two samples are positive with the same bacteria belonging to the oral microbiota, the isolation of which causes possible contamination, and when (ii) only one sample is positive with a specific pathogen bacterium (e.g., Staphylococcus and Mycobacterium species).

Given the importance of microbiological examination in patients with suspected infectious chronic osteomyelitis following an initial bone sample with negative microbiology, some authors suggest repeating biopsies to maximize the microbiological diagnosis yield, as in this case [13].

Bone infections are always serious diseases, and are often difficult to treat, requiring the identification of the causative agent (s) and adequate antibiotic therapy. Due to the high risk of treatment failure, probabilistic or empirical antibiotics, such as those employed in dentistry or oral surgery, have no place in the treatment of bone infections [14]. The choice of antibiotic has been extensively studied, but the antibiotic regimen has not been well defined, although it depends on the organisms being isolated, the spectrum of activity, antibacterial activity and the bone penetration of the antibiotic [12]. Many treatments with fluoroquinolones, rifampicin, clindamycin and cotrimoxazole (trimethoprim/sulfamethoxazole, TMP/SMX) have been reported [15]. In the present case, a combination of levofloxacin and trimethoprim/sulfamethoxazole (TMP/SMX) was used for 6 months.

Levofloxacin is a fluoroquinolone antibiotic, suitable in the treatment of bone infections, due to its high bone penetration; has a persistent antibiotic effect on bone tissue due to its ability to bind to calcium in the inorganic bone matrix; tends to have a lower minimum inhibitory concentration for Gram-positive pathogens; and has a good tolerability within its safety profile. TMP/SMX is a traditional antibiotic with bactericidal activity, and excellent oral bioavailability, and can penetrate bone tissues, making this drug an attractive option for treating chronic osteomyelitis [16]. The main advantage of the combination therapy was to reduce the possibility of selecting resistant bacterial strains [12].

The duration of antibiotic treatment is also an important determinant of therapeutic success. Although an optimal duration of antimicrobial treatment for chronic osteomyelitis has not been well defined, many studies suggest the use of prolonged antibiotic therapy (at least 6 months) [10,12], although other studies support the use of short antibiotic regimens, possibly reducing the emergence of resistant bacteria [15,17]. Beyond 6 months, antibiotic therapy can be individualized based on the clinical and radiologic response, and laboratory tests. In this case, 6 months of antibiotics treatment was sufficient considering the lack of clinical signs of relapses (2-year follow-up), the complete radiographic resolution on CBCT and the normalization of CRP and leukocyte count. Due to the lack of sequestrum, no surgical debridement was necessary, and no additional therapy, such as hyperbaric oxygen therapy, was used.

## 4. Conclusions

The identification of the causative pathogen should be a priority in the diagnosis of chronic osteomyelitis of the jaw. Culture time is crucial, and MALDI-TOF is a helpful tool for pathogen identification. Management within a multidisciplinary team (oral and maxillofacial surgeons, microbiologists, and internists with competence in infectiology) is widely recommended.

## Data Availability

Not applicable.
